# Risk Perception Measurement and Influencing Factors of COVID-19 in Medical College Students

**DOI:** 10.3389/fpubh.2021.774572

**Published:** 2021-11-23

**Authors:** Shangren Qin, Mengqiu Zhou, Ye Ding

**Affiliations:** ^1^School of Public Health, Hangzhou Normal University, Hangzhou, China; ^2^School of Public Health, Hangzhou Medical College, Hangzhou, China

**Keywords:** COVID-19, risk perception, college students, influencing factors, cross-sectional study

## Abstract

**Purpose:** In China, the coronavirus disease 2019 (COVID-19) pandemic has been under control and entered the normal prevention and control stage. For medical college students, many studies have analyzed their knowledge, risk perception, and prevention behaviors of COVID-19, but only a few pieces of research explore the content structure of COVID-19 risk perception and the influencing factors. This study measured the students' risk perception of COVID-19 and its dimensions and analyzed the influencing factors of risk perception among them.

**Methods:** The online questionnaire survey was conducted at Hangzhou Medical College in Zhejiang Province among undergraduates and junior college students. A scale was formulated to precisely measure and analyze the COVID-19 risk perception among medical college students. The factors affecting the COVID-19 risk perception in medical college students were analyzed using the multivariate linear regression model.

**Results:** A total of 810 medical students participated in the survey. Results show that COVID-19 risk perception among medical college students was divided into four dimensions: perceived health threat, perceived severity, perceived controllability, and perceived infection possibility. The results showed that income, education, major, and COVID-19 knowledge were the important factors affecting the COVID-19 risk perception of medical college students. Related factors have different influences on the various dimensions of COVID-19 risk perception. COVID-19 knowledge was significantly related to all dimensions of risk perception.

**Conclusion:** This study evaluates the content structure of medical college students' risk perception of COVID-19 precisely and related influencing factors. It is necessary to grasp the risk perception, prevention, and control behaviors of medical college students of different backgrounds, education levels, and majors. Further knowledge training should be conducted for students majoring in clinical medicine, especially the pandemic prevention and control measure training to enhance their sense of security at work.

## Introduction

At the end of 2019, coronavirus disease 2019 (COVID-19) was discovered and has prevailed. It turns out that this infectious disease can pose a severe threat to human health and cause tremendous economic losses. COVID-19 is different from the previous infectious diseases in its extremely strong infectivity, which doubles that of seasonal flu (1). This virus can spread primarily through routes like respiratory droplets and close contact (2). In January 2020, China incorporated COVID-19 into the *People's Republic of China Law on the Prevention and Treatment of Infectious Disease* as a category B notifiable disease but managed it as a category A infectious disease. It shows that although China designates it as a B-class infectious disease, it pays particular attention to its prevention and control. That is, it is managed as a Class A infectious disease. At present, the COVID-19 pandemic in China has been under control and entered the normal prevention and control stage.

The control of infectious diseases depends on the cooperation of the general population and the improvement of personal protection (3). Related research discovers that risk perception can affect the willingness and motivation of people to take prevention measures (4). Belonging to the psychological category, risk perception emphasizes the perception, and understanding of an individual's various objective external risks (5), which is the foundation of people's response behaviors to emerging public health issues (6). Moreover, it plays a central role in shaping health prevention and control behaviors (7, 8) and is a major predictive factor for preventing and alleviating irrational behaviors. For instance, people with low-risk perception tend to adopt risk-taking behavior or reduce prevention behavior (9).

College students are one of the most active populations in China. In many cases, Chinese colleges teach in large classrooms, and the contact between students is restricted. Chinese colleges are generally located in metropolitan areas, so students have more free time to move to urban areas than workers. In particular, medical college students are the medical reserves who are the most likely to participate in medical behaviors. Therefore, understanding their risk perception will effectively guide them to correctly cope with and prevent secondary hazards induced by improper behavior and response. For example, some studies discovered that the high COVID-19 risk perception in college students increases their depression, anxiety, and pressure (10). However, the experience of great psychological stress will adversely influence the education of medical college students and overall psychological health (11). Some scholars analyze the COVID-19 risk perception and related behaviors among medical college students, like the relationship between risk perception among overseas students and the homecoming behavior (12). Further, some studies analyze the COVID-19 knowledge, risk perception, and prevention behavior of medical college students (knowledge, attitudes, and practices; KAP); for instance, research by medical college students in developed countries like Italy (13), and research by medical college students in developing countries like Egypt (14) and Libya (15).

However, the current research on COVID-19 risk perception among medical college students focuses on analyzing overall COVID-19 risk perception and the KAP. Typically, the measure of risk perception is only manifested by individual problems in the questionnaire rather than systemic measures, and few studies have explored the content and structure of COVID-19 risk perception. Moreover, some studies examine the distribution of COVID-19 knowledge and risk perception among medical college students. But little research directly analyzes the influencing factors of COVID-19 risk perception among medical college students.

Therefore, this study developed a standardized scale to intensively measure the content and structure of COVID-19 risk perception among medical college students, compared the difference in COVID-19 risk perception and its relationship with COVID-19 knowledge, and explored the influencing factors of COVID-19 risk perception among medical college students. The implementation of this study will provide research support for the prevention and control practices of the pandemic in the Chinese universities and other regions in the world. Although concerted efforts are maintaining the pandemic at low levels in China compared to other world areas, the worldwide COVID-19 epidemic is still not over. The pandemic control still should be strengthened in colleges. Therefore, we hope our work will be helpful for the prevention and management of the pandemic among Chinese students and other regions in the world.

## Materials and Methods

### Research Design and Data Source

This study was a cross-sectional study aiming to explore the measuring method of COVID-19 risk perception among medical college students, to evaluate their knowledge, and to analyze the influencing factors. The study population of this article is the medical college students from the Hangzhou Medical College. This study first measured the COVID-19 risk perception level among medical college students in the form of scale (for the detailed development process of this scale, see below “3.1 Measure of COVID-19 risk perception among medical college students”). Second, we analyzed the distribution of each dimension of COVID-19 risk perception among different student populations and examined the association of COVID-19 knowledge with each dimension of risk perception. Finally, a multivariate linear model was adopted to analyze the influencing factors of COVID-19 risk perception among medical college students through the hierarchical regression method.

From June 8 to 28, 2020, a cross-sectional survey was conducted at Hangzhou Medical College of Zhejiang Province. Hangzhou Medical College is the only medical college affiliated with the Healthcare Commission of Zhejiang Province. It has about 7,000 full-time students in 2020. There are 15 undergraduate medical majors (like clinical medicine, preventive medicine, pharmacy, and nursing) together with nine junior medical college majors (such as medical laboratory technology and health information management) for the time being. Notably, it has a profound influence in Zhejiang Province. The anonymous questionnaire (when filling out the questionnaire, respondents' names do not need to be filled in and saved) was established using an online survey platform (Wen Juan Xing, wjx.cn). Then the questionnaire was forwarded through the DingDing app and Wechat app. The participant inclusion criteria, the purpose of the survey, and information confidentiality were stated at the beginning of the questionnaire to obtain participants' informed consent. Participants were asked to fill out the questionnaire after the consent. Participants were encouraged to share the questionnaire with their friends or classmates in the same school. For preventing duplication, one student was just asked to fill in the questionnaire once (each student is set to have only one opportunity to fill in this questionnaire). Therefore, a snowball sampling was used, and a total of 810 valid questionnaires were collected at last (successfully filled out and submitted questionnaires are valid questionnaires, incomplete questionnaires cannot be submitted).

### Research Variables and Measures

#### Dependent Variables and Measures

The COVID-19 risk perception level among medical college students was the dependent variable of this paper, which was measured in the form of a scale. The detailed items are presented in Table 1. Each tested student gave a score for each item based on the subjective degree of agreement, where five points stood for “strongly agree,” four indicated “relatively agree,” three stood for “can't say agree or not,” two represented “slightly disagree,” and one showed “totally disagree.” Later, this study adopted exploratory factor analysis (EFA) to reduce the scale contents' dimensionality. Finally, four dimensions were generated from the COVID-19 risk perception scale, and the sum of all indicators of each dimension was the level of that dimension.

**Table 1:** Factor analysis for the risk perception of coronavirus disease 2019 (COVID-19).

**Factor**	**Items**	**Components (rotation factor loadings)**				**Rotation sums of squared loadings**		
		**Factor1**	**Factor2**	**Factor3**	**Factor4**	**Total**	**% of variance**	**Cumulative %**
Perceived health threat of the COVID-19 epidemic	1. Once infected with COVID-19, my health will be severely affected.	0.833				4.209	35.075	35.075
	2. Even a person is cured of COVID-19, there will be sequelae.	0.775						
	3. Once the COVID-19 breaks out again, it will negatively impact the society immediately.	0.692						
Perceived severity of the COVID-19 epidemic	4. I think this COVID-19 epidemic is very widespread.		0.869			1.854	15.453	50.527
	5. The COVID-19 is easy to spread out.		0.864					
	6. I think the COVID-19 epidemic is more serious than previous infectious diseases (SARS, avian flu).		0.676					
Perceived controllability of the COVID-19 epidemic	7. I think the local status of COVID-19 is very serious.			0.788		1.182	9.85	60.378
	8. I think the epidemic and spread of COVID-19 is difficult to control.			0.678				
	9. I think the COVID-19 is very difficult to treat.			0.640				
Perceived infection possibility of COVID-19	10. I am very likely to be infected with COVID-19.				0.502	1.009	8.412	68.790
	11. As long as I have been in contact with the items of a COVID-19 patient, I may be infected.				0.866			
	12. As long as I am in the same space with a COVID-19 patient, I may be infected by him.				0.854			

*Extraction method:principal components; Rotation method: Varimax*.

#### Independent Variables

Independent variables were classified into three categories, such as demographic characteristics, major, and COVID-19 knowledge.

Demographic information of students in our questionnaire includes age, sex, education (undergraduate or junior college), student origin (Zhejiang Province or others), and monthly per capita household income.

Major is a category that colleges divide their studies into according to the science division in China, and usually, a college student only has one major. In this article, majors of medical college students were divided into four types based on study faculty, such as clinical medicine, public health, nursing, and medical technology (laboratory technology and imaging technology included).

COVID-19 knowledge topics. For medical college students, their mastery levels of knowledge on COVID-19 were assessed by five single-choice questions, such as “(1) Do asymptomatic COVID-19 carriers show infectivity? (2) How long should the segregation period be after close contact with the patients of COVID-19? (3) For the time being, what is the infectious disease classification grade and management grade of COVID-19? (4) Which is not the main transmission route of COVID-19 found currently? (5) Which disinfection means fail to deactivate COVID-19 efficiently?” The wrong answer was rated as 0 points for every question, and the correct answer as 1 point.

### Statistical Methods

The EFA method was utilized for dimensionality reduction analysis of the COVID-19 risk perception scale. In addition, a *t*-test was used to analyze the differences of each dimension of risk perception between variables, as well as the COVID-19 knowledge.

Each dimension of COVID-19 risk perception among medical college students was used as the dependent variable. The influencing factors of risk perception level among medical college students were analyzed using a multivariate linear regression model. In addition, the hierarchical regression method was used to incorporate the demographic characteristics, major categories, and COVID-19 knowledge variables into the equation step by step to obtain model 1, model 2, and model 3, respectively. Among them, the equation was not significant after incorporating COVID-19 knowledge into the model for the perceived severity dimension, so model 3 was excluded, as shown in **Table 4**.

When using the multivariate linear regression model, model estimation was performed by the weighted least square method to solve the problem heterogeneity of variance in some models. Meanwhile, the variance inflation factor (VIF) test was employed to detect the collinearity problem. It was found that the VIF value of each model was close to two, suggesting the absence of collinearity problem in the model. Moreover, the autocorrelation problem was detected by the Durbin–Watson test (D–W test). It was discovered that the DW value of each model was close to 2, indicating that there was no autocorrelation.

All statistical tests were two-sided, and *p* < 0.05 indicated statistical significance. Data were analyzed using R software (version 3.6.3).

## Results

A total of 810 medical students participated in the survey, with an average age of 19 years. Approximately 68.69% of them were female. Most of them were from Zhejiang Province (80.12%).

### Measure of COVID-19 Risk Perception Among Medical College Students

This study developed a scale to measure the COVID-19 risk perception among medical college students, which contained 12 items, as shown in Table 1.

The EFA method was conducted for the dimensionality reduction analysis of this scale. First of all, the Kaiser–Meyer–Olkin (KMO) test and Bartlett test of sphericity were performed to test whether the data were suitable for factor analysis. KMO was calculated to be 0.796 (>0.5); besides, EFA was validated to be applicable by the Bartlett test of sphericity (χ^2^: 3,464.209, *p* < 0.001). Later, EFA was performed on the data, and factors were extracted by principal component analysis (PCA). According to Table 2, all the 12 items were divided into four dimensions, accounting for 68.790% variance. As suggested by item contents and literature (16), dimensions one to four were classified as “perceived health threat,” “perceived severity,” “perceived controllability,” and “perceived infection possibility,” respectively.

**Table 2:** Univariate analysis of the risk perception of COVID-19 among medical college students with different socio-demographic factors.

**Socio-Demographic factors**	**Number (%)**	**Perceived health threat**		**Perceived severity**		**Perceived controllability**		**Perceived infection possibility**	
		**Score^a^**	***P***	**Score^a^**	***P***	**Score^a^**	***P***	**Score^a^**	***P***
**Sex^b^**									
Male	252 (31.11)	10.85 ± 2.85	0.454	13.37 ± 2.25	0.561	9.78 ± 2.95	0.202	8.92 ± 3.12	0.297
Female	558 (68.89)	10.70 ± 2.48		13.28 ± 1.77		9.51 ± 2.40		8.68 ± 2.72	
**Province^b^**									
Zhejiang	649 (80.12)	10.81 ± 2.61	0.145	13.38 ± 1.83	0.086	9.57 ± 2.66	0.578	8.92 ± 2.84	<0.001^***^
Others	161 (19.88)	10.48 ± 2.54		13.04 ± 2.32		9.68 ± 2.25		8.06 ± 2.78	
**Education level^b^**									
Junior college	334 (41.23)	10.90 ± 2.35	0.159	13.06 ± 2.13	0.003^**^	9.41 ± 2.65	0.101	9.11 ± 2.64	0.002^**^
Undergraduate	476 (58.77)	10.64 ± 2.76		13.49 ± 1.77		9.72 ± 2.54		8.50 ± 2.97	
**Household monthly income per capita (RMB,Yuan)^c^**									
<2,000	126 (15.56)	11.04 ± 2.43		13.40 ± 1.91		9.93 ± 2.49		9.06 ± 2.63	
2,000–3,999	221 (27.28)	10.61 ± 2.40	0.005^**^	13.07 ± 1.96	0.022^*^	9.38 ± 2.33	0.017^*^	8.66 ± 2.82	0.023^*^
4,000–5,999	222 (27.41)	10.31 ± 2.47		13.18 ± 1.88		9.32 ± 2.57		8.41 ± 2.66	
6,000–7,999	102 (12.59)	10.91 ± 2.72		13.42 ± 1.80		9.50 ± 2.53		8.54 ± 2.88	
≥8,000	139 (17.16)	11.28 ± 3.03		13.73 ± 2.04		10.12 ± 3.02		9.33 ± 3.27	
**Major^c^**									
Public health	169 (20.86)	10.71 ± 2.82		13.15 ± 1.91		9.65 ± 2.58		8.69 ± 2.86	
Clinical medicine	318 (39.26)	10.74 ± 2.81	0.996	13.65 ± 1.68	<0.001^***^	9.70 ± 2.68	0.619	8.55 ± 3.05	0.247
Medical technology	155 (19.14)	10.77 ± 2.42		13.27 ± 2.20		9.52 ± 2.57		8.89 ± 2.85	
Nursing	168 (20.74)	10.77 ± 2.10		12.85 ± 2.05		9.39 ± 2.43		9.07 ± 2.40	

a *Mean ± Standard deviation.*

b *t-test.*

* *p < 0.05,*

** *p < 0.01,*

*** *p < 0.001.*

c variance analysis. ^*^p < 0.05, ^**^p < 0.01, ^***^p < 0.001.

Calculating Cronbach?s alpha coefficient was used to test the reliability of the questionnaire. The Cronbach's alpha coefficient for each dimension is 0.758, 0.764, 0.690, and 0.751. A reliability index ≥0.6 is considered acceptable (17). So, this indicates that the four factors (dimensions) of the COVID-19 risk perception construct are reliable.

The score of each item in each dimension was summarized as the score of that dimension. The median scores of the four COVID-19 risk perception dimensions were 11, 14, 9, and 9 points, respectively. Obviously, the overall levels of perceived health threat and perceived severity were higher.

### Differences in COVID-19 Risk Perception Among Medical College Students and the Distribution

Table 2 exhibits the student background characteristics and distribution of each dimension of COVID-19 risk perception. A total of 810 participants were enrolled in the present survey. Among them, 68.89% were female students. Most of them came from Zhejiang Province (80.12%), 58.77% were undergraduates, while ￥2,000–3,999 and ￥4,000–5,999 (27.28 and 27.41%) were the dominant per capita family monthly incomes. As shown in Table 2 from the perspective of the origin of students, students from Zhejiang Province had a higher level of perceived infection possibility than students from other provinces, and the difference was statistically significant. Regarding education level, differences in “perceived severity” and “perceived infection possibility” dimensions between undergraduates and junior college students were statistically significant. Besides, among students with different per capita family monthly incomes, differences in all dimensions of risk perception were statistically significant. Further, there were differences in COVID-19 risk perception among medical college students of different majors, and students of clinical major had a relatively higher level of perceived severity.

### COVID-19 Knowledge and Risk Perception Assessment Among Medical College Students

Different from the COVID-19 measuring methods in previous studies, this study measured the COVID-19 risk perception and knowledge levels among medical college students from various dimensions and analyzed the association between the two. After calculation, the median score of the COVID-19 knowledge among the medical college students was three points. Table 3 exhibits the COVID-19 knowledge and distribution of the risk perception dimensions among medical college students. There were differences in the mastering of different knowledge items and COVID-19-related dimension risk perception. For instance, students who understood the infectivity of asymptomatic patients had a higher level of perceived severity. Students who understood the COVID-19 infectious disease grade classification and risk management grade showed lower levels of perceived health threat, perceived controllability, and perception of infection probability, and the differences were statistically significant.

**Table 3:** Univariate analysis of the risk perception of COVID-19 among college students with different knowledge.

**Knowledge of COVID-19**	**Right/wrong**	**Perceived health threat score^a^**			**Perceived severity score^a^**			**Perceived controllability score^a^**			**Perceived infection possibility score^a^**		
		**Right**	**Wrong**	***P*** **^b^**	**Right**	**Wrong**	***P*** **^b^**	**Right**	**Wrong**	***P*** **^b^**	**Right**	**Wrong**	***P*** **^b^**
1. Infectiousness of asymptomatic patient	785/25	10.77 ± 2.61	9.88 ± 2.26	0.091	13.34 ± 1.92	12.32 ± 2.06	0.009^**^	9.59 ± 2.60	9.52 ± 2.31	0.889	8.75 ± 2.86	8.84 ± 2.58	0.877
2. Medical observation and isolation time	761/49	10.74 ± 2.59	10.82 ± 2.80	0.845	13.33 ± 1.88	12.94 ± 2.62	0.305	9.58 ± 2.56	9.84 ± 2.93	0.494	8.74 ± 2.82	8.98 ± 3.31	0.566
3. Disease classification and management grade in China	374/436	10.45 ± 2.52	11.00 ± 2.65	0.003^**^	13.41 ± 1.75	13.22 ± 2.08	0.178	9.39 ± 2.49	9.76 ± 2.66	0.040^*^	8.37 ± 2.68	9.08 ± 2.95	<0.001^***^
4. Main transmission route	547/263	10.64 ± 2.57	10.97 ± 2.66	0.095	13.39 ± 1.78	13.14 ± 2.22	0.122	9.48 ± 2.52	9.82 ± 2.72	0.084	8.76 ± 2.78	8.74 ± 3.00	0.915
5. Anti-virus measures	532/278	10.57 ± 2.68	10.84 ± 2.55	0.161	13.45 ± 1.78	13.24 ± 2.01	0.129	9.49 ± 2.45	9.65 ± 2.65	0.401	8.58 ± 2.70	8.84 ± 2.92	0.219

a *Mean ± standard deviation.*

b *t-test.*

* *p < 0.05,*

** *p < 0.01,*

*** *p < 0.001*.

### Influencing Factors of COVID-19 Risk Perception Among Medical College Students

Table 4 presents the multivariate linear regression results of the influencing factors of COVID-19 risk perception among medical college students. Compared with men, women showed a low level of the perceived infection possibility dimension. But after incorporating the COVID-19 knowledge factor into the model, the sex factor was no longer statistically significant. Compared with students in Zhejiang Province, medical college students from other provinces showed low levels of perceived severity and perceived infection possibility. Still, after incorporating the COVID-19 knowledge factor into the model, the influence of the origin of the student factor on the perceived severity was no longer statistically significant. However, its impact on the perceived controllability was still statistically significant.

**Table 4:** Multiple linear regression of the risk perception of COVID-19 among medical college students.

**Socio-Demographic factors**	**Perceived health threat**			**Perceived severity**			**Perceived controllability**		**Perceived infection possibility**		
	**Model 1**	**Model 2**	**Model 3**	**Model 1**	**Model 2**	**Model 3**	**Model 1**	**Model 2**	**Model 1**	**Model 2**	**Model 3**
	**β^a^**	**β^a^**	**β^a^**	**β^a^**	**β^a^**	**β^a^**	**β^a^**	**β^a^**	**β^a^**	**β^a^**	**β^a^**
**Sex**											
Male (ref)											
Female	−0.172	−0.145	−0.077	0.008	0.122	0.083	−0.171	−0.151	−0.442^*^	−0.475^*^	−0.3773
**Province**											
Zhejiang (ref)											
Others	−0.293	−0.289	−0.344	−0.380^*^	−0.396^*^	−0.356	0.103	0.0002	−0.902^***^	−0.987^***^	−1.089^***^
**Education level**											
Junior college (ref)											
Undergraduate	−0.295	−0.389	−0.302	0.441	0.110	0.055	0.273	0.373	−0.628^**^	−0.420	−0.342
**Household monthly income per capita(RMB,Yuan)**											
<2,000 (ref)											
2,000–3,999	−0.510	−0.514	−0.522^*^	−0.379	−0.383	−0.365	−0.496	−0.586^*^	−0.498	−0.449	−0.336
4,000–5,999	−0.872^**^	−0.874^**^	−0.860^**^	−0.297	−0.295	−0.301	−0.636^*^	−0.619^*^	−0.830^**^	−0.818^**^	−0.783^**^
6,000–7,999	−0.373	−0.376	−0.385	−0.104	−0.132	−0.122	−0.372	−0.478	−0.789^*^	−0.728	−0.635
≥8,000	0.136	0.133	0.127	0.206	0.163	0.168	0.190	0.148	0.076	0.148	0.194
**Major**											
Public health (ref)											
Clinical medicine		0.214	0.271		0.516^**^	0.498^*^		−0.024		0.021	0.136
Medical technology		0.099	0.111		0.291	0.297		−0.141		0.376	0.470
Nursing		0.032	0.026		−0.196	−0.189		0.031		0.357	0.336
**Knowledge of COVID-19**			−0.263^**^			0.154^*^		−0.278^**^			−0.257^*^
*R^2^*	0.026	0.026	0.036	0.031	0.043	0.049	0.019	0.029	0.044	0.048	0.056
Adjusted *R^2^*	0.017	0.014	0.022	0.022	0.031	0.036	0.011	0.016	0.036	0.036	0.043
*F*-value	3.01	2.17	2.68	3.61	3.62	3.71	2.24	2.18	5.31	4.00	4.34
*P*-value of the model	0.004^**^	0.018^*^	0.002^**^	<0.001^***^	<0.001^***^	<0.001^***^	0.030^*^	0.013^*^	<0.001^***^	<0.001^***^	<0.001^***^
VIFmax	1.953	2.437	2.410	2.056	2.065	2.118	2.056	2.065	2.056	2.437	2.313
DW value	2.037	2.036	2.021	1.858	1.882	1.894	1.880	1.931	1.901	1.886	1.862
Heteroscedasticity *P*-value	0.684	0.355	0.467	0.053	0.077	0.100	0.631	0.090	0.059	0.118	0.066

a *Multiple linear regression*

* *p < 0.05,*

** *p < 0.01,*

*** *p < 0.001*.

From the perspective of education, medical college students at the undergraduate level showed a low level of perceived infection possibility compared with junior college students. But after incorporating the major factor and COVID-19 knowledge factor into the model, the education factor was no longer statistically significant.

The per capita family monthly income was also related to the COVID-19 risk perception among medical college students. Compared with students with the per capita family monthly income below ￥2,000, those with the per capital family monthly income of ￥4,000–5,999 showed low levels of perceived health threat (model 1, model 2, and model 3 in the table), perceived controllability (model 1 and model 2 in the table) and perceived infection possibility (model 1, model 2, and model 3 in the table). After incorporating demographic characteristics, major and COVID-19 knowledge into the equation, and the differences were still statistically significant. However, the influence of the income factor on the perceived severity was not statistically significant.

The perceived severity was associated with the specific major of the students. The student major factor was incorporated into the model. The results suggested that, compared with medical college students of public health major, those of clinical medicine major had a high level of perceived severity, and the difference was of statistical significance.

After incorporating the COVID-19 knowledge factor into the model, it was discovered that this factor was associated with each dimension of the COVID-19 risk perception among medical college students. To be specific, a higher COVID-19 knowledge level indicated the lower levels of perception of pandemic heath threat (model 3), perceived controllability (model 2), and perceived infection possibility (model 3), whereas the higher level of perceived severity (model 3).

## Discussion

Research on people's risk perception contributes to control pandemic transmission. Related studies have analyzed the public panic over COVID-19 risk perception (18) and the pandemic-induced uncertainty, alarm, and sadness (19). However, there is little in-depth research on the risk perception among Chinese medical college students. Therefore, it is important to understand the COVID-19 risk perception among Chinese medical college students and the related influencing factors.

This study treated medical college students as the respondents and systemically measured the manifestation of the risk perception concept in the COVID-19 event through scale. Previous related research on the risk perception measure is quite simple. It can be seen that these measures of the risk perception of infectious diseases are too simple, and too few questions cannot measure the internal structure of risk perception. For instance, research on the impact of public risk perceptions on protective behaviors in the United States and South Korea, compiled 4 questions to measure risk perceptions from the perspectives of perception fragility and perception severity (20). Some scholars investigate the risk perception of H1N1 in 2009 among the Australian public by four questions from the perspective of risk severity (21). However, the risk perspective is an abstract psychological concept. The measurement by the few questions cannot reflect the complexity of phenomenon complexity, which is not precise enough and is to the disadvantage of grasping the variable nature (22), with poor stability and effectiveness. Consequently, this study measured the COVID-19 risk perception among medical college students by scale, which partially compensated for the aforementioned drawbacks.

Further, this study measured the COVID-19 risk perception among medical college students from four dimensions and discovered the more complex relationship between the COVID-19 knowledge level and risk perception. Such result is not completely consistent with the result that the higher COVID-19 knowledge level among medical college students is associated with the higher risk perception pointed out in related research (5). These conclusions may not be contrary, since our study further classified the content and dimension of risk perception. In some dimensions, such as the perceived severity dimension, the knowledge level was positively correlated with the risk perception. In other aspects, such as perception of health threat, perceived controllability, and perceived infection possibility, knowledge level was negatively correlated with the risk perception level. Thus, there may be differences between knowledge and risk perception from different risk perception dimensions.

This study suggested that the perceived infection possibility was lower in women, which was consistent with the risk perception research among the Iran medical college students (this may mean that the women in this study were more aware of the importance of COVID-19) (23). Nonetheless, some research discovers that the risk perception in women may be higher, like the risk perception of severe acute respiratory syndrome (SARS) (24) and Middle East respiratory syndrome (25). Such inconsistency is possible because this study further classified COVID-19 risk perception into four dimensions. When not considering knowledge and major, women had a lower level of perception of infectivity. Clearly, the detailed internal dimensions of risk perception should be combined when analyzing the influence of sex on risk perception.

Relevant research suggests that COVID-19 risk perception is related to family income. The higher income is related to the lower perception of death risk because the rich people can afford the disease treatment (26), while those with low incomes are not willing to stay at home since they have to go out to work in the economic recession. Our findings were consistent with these studies, which showed that compared with low-income families, studies from the average and above monthly income families had low levels of the perceived health threat, perceived controllability, and perceived infection possibility. This might be related to the job types of low-income families. According to the studies mentioned above, people with low income have to work in occupations with high exposure risk, which together with the imperfect risk prevention measures, results in a high-risk perception level.

Related research discovers that attainment/education level is related to risk perception (27). This study found that, compared with junior college students, the undergraduates had lower perceived infection possibility when the major factor was not considered. This is inconsistent with related research. For instance, some scholars discover that the lower attainment/education level is related to the lower risk perception (28). This may be caused by inconsistent measuring methods. Unlike other studies that measure the attainment levels of students by grade, this study measured the education level by undergraduates and junior college students. Generally, the attainment level of junior college students is lower than that of undergraduates in China. Indeed, these two methods of measuring attainment levels are somewhat different. Moreover, this study further classified the measures of risk perception contents, which was also an important cause, since this study discovered that education level only affected the perceived infection possibility, but not other dimensions.

Major may be associated with the risk perception. For instance, some studies discover that students of medical majors have lower risk perception levels than those of non-medical majors (29, 30). This study further intensively analyzed the risk perception among students with different majors, such as public health, clinical medicine, medical technology, and nursing. It was discovered that compared with medical college students of public health majors, those of clinical majors had a higher perception of COVID-19 severity. This may be because that the clinic-oriented students get more related knowledge (31). Moreover, as future doctors, clinic-oriented medical college students are more likely to be directly exposed to the medical environment.

Certain limitations should also be noted in this study. First of all, concerning the assessment of COVID-19 knowledge among medical college students, the COVID-19 knowledge topics developed in this study were not enough. Second, in terms of samples, this study only investigated students from one medical college, and the research results might not be powerful to deduce all medical college students. The investigation was quickly completed in a short period when students of all majors were in school due to the upcoming final exam. Therefore, there is a narrow time window for our study. Moreover, some other valuable influencing factors were not incorporated, like the students' physical condition, medical insurance status, psychological characteristics, and social capital, which might be the important factors affecting risk perception. These factors may be incorporated into further research for analysis.

In summary, this study measures the COVID-19 risk perception among medical college students by means of scale and embodies risk perception into different internal dimensions, and also discovered that the related factors have different influences on each dimension of COVID-19 risk perception.

There are some insights for future research regarding this topic. A recommendation for future research is to broaden valuable influencing factors affecting risk perception among medical college students, such as physical condition and medical insurance status of students. In addition, based on the findings of this study, the relationship between different dimensions of COVID-19 risk perception and prevention and control behaviors can be further explored in the future.

## Conclusions and Implications

This study intensively measures the COVID-19 risk perception and its dimensions among medical college students and analyzes the influencing factors of COVID-19 risk perception. The COVID-19 risk perception can be divided into four dimensions, namely, “perception of pandemic heath threat,” “perceived severity,” “perceived controllability,” and “perceived infection possibility.” The analysis results suggest that income, education level, major, and COVID-19 knowledge are the important factors affecting COVID-19 risk perception among medical college students.

This study further promotes the related academic theoretical research. First, this study measures the COVID-19 risk perception among medical college students by means of scale and embodies risk perception into different internal dimensions, which partially compensates for the drawbacks in relevant research. Because a single measurement index lacks preciseness and measuring stability. Second, this study also discovered that related factors have inconsistent influences on each dimension of COVID-19 risk perception. Consequently, the detailed internal dimensions and related factors of risk perception should be combined for the analysis when analyzing risk perception.

This study also facilitates to promote the pandemic prevention and control practices. We found the pandemic risk perception was associated with the prevention behavior. Therefore, in the future normal pandemic prevention and control process, risk perception and prevention and control behaviors among medical college students of different backgrounds should be grasped. Moreover, further health education training should be carried out. All in all, it is necessary to help these students build a positive attitude toward COVID-19.

## Data Availability Statement

The raw data supporting the conclusions of this article will be made available by the authors, without undue reservation.

## Ethics Statement

This study was approved by the Ethics Committee of Hangzhou Medical College (Ethics code: LL2020-40). The patients/participants provided their written informed consent to participate in this study.

## Author Contributions

SQ conceived the idea and design of this study and he also dealt with data analysis. MZ further improved the quality of manuscript writing. YD performed the survey and wrote the manuscript. All authors contributed to the article and approved the submitted version.

## Funding

This study was funded by Scientific Research Fund of Zhejiang Provincial Education Department (Grant no. Y201737052), Medical and Health Technology Plan Project of Zhejiang Province (Grant no. 2022RC126), Zhejiang First-class Discipline Public Health and Preventive Medicine (Grant no. 6310058042), and the National Natural Science Foundation of China (Grant no. 71704042). The financial sponsor played no role in the design of the study and collection, analysis, and interpretation of data and in writing the manuscript.

## Conflict of Interest

The authors declare that the research was conducted in the absence of any commercial or financial relationships that could be construed as a potential conflict of interest.

## Publisher's Note

All claims expressed in this article are solely those of the authors and do not necessarily represent those of their affiliated organizations, or those of the publisher, the editors and the reviewers. Any product that may be evaluated in this article, or claim that may be made by its manufacturer, is not guaranteed or endorsed by the publisher.
